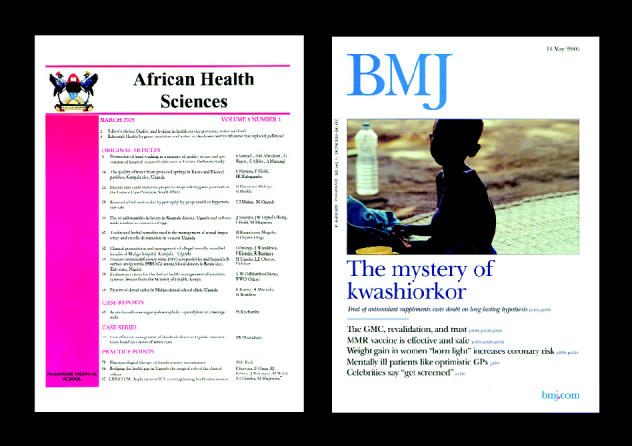# Global Collaboration Gives Greater Voice to African Journals

**Published:** 2005-07

**Authors:** Tanya Tillett

Peer-reviewed journals are a vital source of information exchange for researchers and clinicians in the medical and environmental health arena. The timely publication of credible research is integral to advancing the realm of knowledge in any given topic area. Many Northern Hemisphere journals have access to a range of resources to aid in timely publication: a database of potential peer reviewers, adequate staff and tools to produce each issue, and the funds to ensure publication. However, journals in developing regions such as sub-Saharan Africa usually don’t have these same resources. As a result, publication can be erratic, and important information may not reach the people who need it most. Now a multinational partnership of editors from science and medical journals, including *EHP*, is working to help African journals gain a greater voice on the scientific publishing stage.

## Impediments to Publication

Considering that many of the least-developed, lowest-income countries in the world are located in sub-Saharan Africa, it’s not surprising that many assets that are taken for granted in the offices of well-established Northern Hemisphere journals are unavailable to journals in that region. Currently, most African medical journals are funded by academic institutions or professional organizations that usually have only extremely limited funding available. Although it is common for the journals to sell subscriptions and advertisement space, only a small amount of income is generated from these sources due to lack of subscribers and advertisers. Most of the journals have minimal staff to coordinate both the review and production processes, and access to technological tools and other equipment and supplies to facilitate the work is not always possible.

The credibility of published research is heavily dependent upon the peer review process, yet the average African journal has only a limited number of manuscript reviewers. Many things factor into this circumstance: difficulty maintaining reviewer anonymity, cultural mores that discourage criticism of more senior researchers, reviewer conflict-of-interest issues, and the lack of an established system for academic or professional recognition of reviewer input.

All these factors together can lead to an irregular publication schedule, which—with the perception that publication in Northern Hemisphere journals will facilitate more rapid career development—can deter local scientists and clinicians from publishing in their national journals. Therefore, manuscript submission rates are low, and research quality cannot be ensured.

Another difficulty facing African journals is dissemination of their content to other parts of the world. Most information published in African journals never leaves its home borders because these journals are largely not included in major bibliographic databases like the National Library of Medicine’s (NLM) MEDLINE. Such databases have criteria—ranging from quality of content to production quality—that must be met before a journal is accepted for indexing. Only 31 of the 4,900 journals indexed by MEDLINE are from Africa, while more than 4,300 are Northern Hemisphere journals.

## Partnerships for Change

To combat the problems faced by journals in Africa, a group of African and Northern Hemisphere medical journal editors met with representatives of other interested organizations at an October 2002 World Health Organization workshop, and created the Forum for African Medical Editors (FAME). Around the same time, representatives from the NLM and the John E. Fogarty International Center (FIC) were discussing how more journals in Africa might be upgraded for acceptance into MEDLINE. In September 2003, respresentatives from the NLM, the FIC, the NIEHS, and nine journals met at the offices of the *British Medical Journal* (now known officially as *BMJ*) to discuss ideas for partnerships. The meeting attendees created the African Medical Journal Editors Partnership Program.

Gerald Keusch, assistant provost for global health at Boston University and former director of the FIC, initiated and participated in the 2003 meeting. As Keusch recounts, the participants deemed the most logical answer to solving the limited capacity dilemma of the African journals to be partnering them with counterparts in the United States and the United Kingdom.

Thus, several partnerships were established at this meeting: *African Health Sciences* (Uganda) was partnered with *BMJ*, *Ghana Medical Journal* was paired with *The Lancet*, *Malawi Medical Journal* was matched with the *Journal of the American Medical Association* (now officially known as *JAMA*), and *Mali Médical* was partnered with the *American Journal of Public Health* (*AJPH*) and *EHP*. All the participating African journals are in countries where active NIH research is ongoing, and the five partnering Northern Hemisphere journals are interested in promoting public health in developing nations around the world. The FIC, the NLM, and the NIEHS contributed funds to start the program. A contract was then awarded to the Council of Science Editors (CSE) to manage the funds for this pilot project [also see *EHP* 112: A858 – A860 (2004)].

Why participate in this partnership? For the African journals, the reasons are fairly obvious. For research publications, being connected to the global information network is key to keeping abreast of and benefiting from the very latest information. A partnership like this one would help stabilize the African journals’ connection to today’s information exchange systems and would aid in the compilation and dissemination of public health information in the developing African countries. This, says Keusch, would allow information of a local nature to be more readily accessed locally, and also would allow the evolution of quality publications that all could take pride in.

The Northern Hemispheres journals will benefit too. Thomas J. Goehl, *EHP*’s editor-in-chief, says that, in addition to participating for purely altruistic reasons, the Northern Hemisphere journals also see this as a unique opportunity to help disseminate research and public health information being generated in Africa. Because developing nations are often exposed to toxicants and pathogens in much greater concentrations and numbers than developed nations, research opportunities abound to learn the basic biology of pathologies that plague both Africa and other countries around the world, he says.

Goehl adds that by creating methods to help developing nations gather and distribute the knowledge they have, industrialized nations can consider those medicinal and traditional procedures when improving their own scientific and medical methods. Thus, a true partnership in research between the developing and industrialized world could ultimately benefit everyone.

However, says Goehl, above all we should be motivated by a duty to protect humanity. “I believe health care should be considered a basic human right,” he says. “If this is true, then we have a moral imperative to provide adequate health care for all people. I hope *EHP* can contribute to this goal by strengthening journals that are needed to disseminate relevant and credible information to practicing medical professionals.”

## Off to a Good Start

Since forming the partnership, the member journals have been involved in several activities. Two training meetings have been held in Africa for local editors and reviewers to develop more effective editorial guidelines for their journals, sharpen writing skills, and improve current manuscript managing processes.

To date, all but one African journal partner have visited the offices of their respective Northern Hemisphere partners. Visits usually last 5–10 days to allow the editors time to get a true sense of how their partner journals operate, and also to give them the opportunity to see how they could adapt operations to fit their own journals’ needs. In the near future, staff from the Northern Hemisphere journals will visit their African counterparts to assist in onsite capacity building.

Supplies needed to facilitate production also are on the way. The NLM has sponsored site visits to each African journal by local technical experts. These experts identified employee training needs and desktop publishing functionality, made hardware and software recommendations, and assessed the journals’ Internet connectivity.

One partner, the *Ghana Medical Journal*, has already received full equipment upgrades. David Indome, site manager for the technical upgrades, is pleased with how well the transition is going there, and sees the technical overhaul as a change that will help the journal operate much more efficiently. Before the upgrade, the journal was using one computer for all aspects of journal production. The computer, an older model, used severely outdated word processing and virus software. With the upgrade, the staff now has a scanner and two computers with more current word processing, bibliography, desktop publishing, and virus software. “Thanks to the hardware and software upgrade, the *Ghana Medical Journal* can now prepare articles much faster than before because they are using programs that are easier, quicker, and more convenient,” says Indome. Reliable Internet connection also allows for much easier interaction between editors, reviewers, and authors.

According to Julia Royall, NLM’s chief of international programs and administrator of the equipment and training portion of the partnership, these technical upgrades will help get all of the journals operating at a more efficient level so they can meet the criteria for inclusion in MEDLINE.

## *EHP, AJPH,* and *Mali Médical*

*EHP* and *AJPH* are also actively working with their partner journal, *Mali Médical*. Operating in Mali’s capital, Bamako, *Mali Médical* was established in 1975 and published up to four issues per year through 1998. Editor-in-chief Siaka Sidibé has been at the helm since 1996, and since 1998 the journal has consistently published four issues each year.

Sidibé says areas needing immediate attention include computer equipment, Internet access, and training for the editors and reviewers. Another challenge facing the journal is printing costs. Although articles are edited and laid out on a strict schedule, the actual timing of printing an issue can vary due to funding issues. *Mali Médical* currently has a volunteer editorial board of seven and a staff of three volunteer editors and one paid part-time support person. The journal currently is not indexed in any of the major bibliographic databases.

The week of 24 September–1 October 2004, Goehl, Sidibé, and *AJPH* editor-in-chief Mary E. Northridge met at the *EHP* offices in Research Triangle Park, North Carolina, to develop a plan of action for the African journal. Of the four African partner journals, *Mali Médical* is the only one from a francophone nation. With *EHP* and *AJPH* both being English-language journals, language is a challenge—especially in some training efforts—but has not dampened the enthusiasm and resolve of the three partners.

*Mali Médical* will soon be equipped with hardware and software, and staff will be trained in their use. Through funding from the partnership, a full-time managing editor will be hired for desktop publishing and office administration. The journal also has a new website, http://www.malimedical.org/, which is currently hosted by *EHP*. The web-site contains articles archived from 2003 to the present, and will eventually be housed on *Mali Médical*’s own server.

Once all the basic necessities for efficient management are gathered, the journal can begin to focus on raising its recognition. Efforts to this end will include co-publication of research articles in *EHP* and *AJPH*, as well as exploration of online manuscript submission and peer review, says Sidibé.

## Looking Ahead

The members of the partnership are forging ahead with their goals. Representatives from each partner journal, the FIC, the NLM, and the CSE met during the May 2005 CSE annual meeting to review the first year’s activities and plan for the upcoming year. All agreed that much progress has been made.

All of the tasks defined by the partnership are being addressed, and the African partners have embraced the project. With funding assistance from the FIC, the NLM, and the NIEHS, the four African journals will copublish review articles on several “neglected” diseases, illnesses that generally affect poor people in poor countries and thus may not garner as much research attention from more affluent nations. These articles will appear in both English and French in each journal’s September 2005 (or equivalent) issue. With help from FAME, they have also been able to develop training sessions for editors and research paper writers.

The partners also refined certain original tasks set for the program. For example, instead of training African journal staff in a breadth of skills, a consensus was reached to develop focused training that concentrates on one aspect of the publication process (such as manuscript handling, or marketing and public relations). This concentrated training will help the African journals obtain skills and strengthen their operations at a more accelerated pace.

The participants also agreed they must emphasize public relations to secure more funding. More funding, in turn, will help the African partners achieve and maintain a more regular publication schedule.

Three of the African journals cannot yet be abstracted in MEDLINE, but they have learned they can still submit their content to PubMed Central for archiving, allowing their articles to be read worldwide. Participation in PubMed Central is open to any life sciences journal that meets NLM’s standards for scientific and editorial quality of its content and technical quality of its digital files. Having stable websites could help the journals publish online on time, increasing their chances acceptance into MEDLINE.

The editors themselves will obtain additional help from ScholarOne, a provider of web-based applications to improve the workflow for scholarly journals. ScholarOne has offered software and training services free of charge for five years for each African journal, so the journals can set up and maintain their own online manuscript submission and review systems. SPI Publisher Services has also offered its services free of charge for five years. This company will convert each journal’s files to XML format, a flexible web-site code required for MEDLINE and PubMed Central that allows for more sophisticated website navigation.

The African Medical Journal Editors Partnership Program began with one good idea shared by editors thousands of miles apart. Through the dedication and enthusiasm of the partners and their supporters, great strides have been made in just a short time. Goehl thinks even greater strides are yet to come. “It is a committed one-on-one partnership,” he says, “that is the key to the success of journal capacity building in the developing world.”

## Figures and Tables

**Figure:**
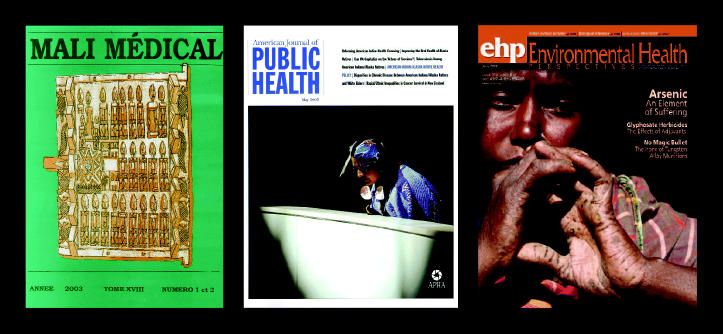


**Figure:**
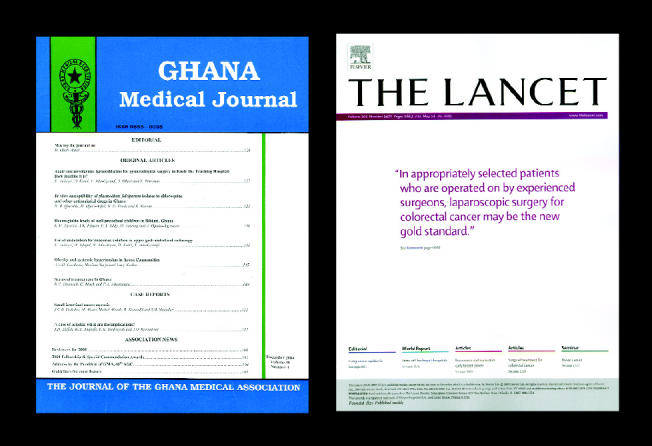


**Figure:**
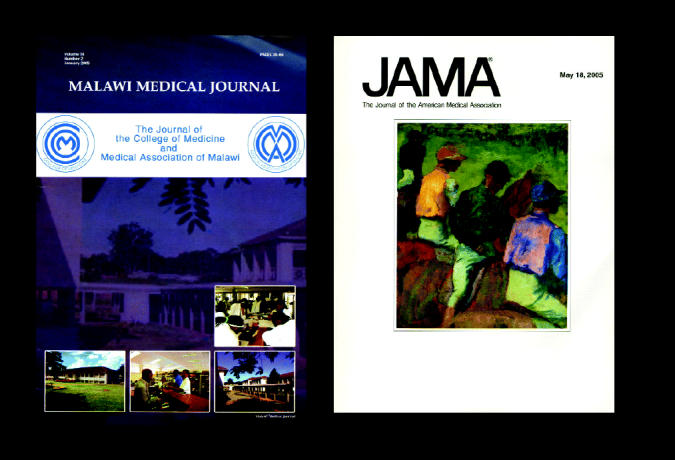


**Figure:**